# Searching for virus phylotypes

**DOI:** 10.1093/bioinformatics/btt010

**Published:** 2013-01-17

**Authors:** François Chevenet, Matthieu Jung, Martine Peeters, Tulio de Oliveira, Olivier Gascuel

**Affiliations:** ^1^Institut de Biologie Computationnelle, LIRMM, UMR 5506 CNRS – Université Montpellier 2, Montpellier, France, ^2^MIVEGEC, CNRS 5290, IRD 224, Universités Montpellier 1 et 2, Montpellier, France, ^3^TransVIHMI, UMI233, IRD – Université Montpellier 1, Montpellier, France, and ^4^Africa Centre for Health and Population Studies, University of KwaZulu-Natal, Durban, South Africa

## Abstract

**Motivation:** Large phylogenies are being built today to study virus evolution, trace the origin of epidemics, establish the mode of transmission and survey the appearance of drug resistance. However, no tool is available to quickly inspect these phylogenies and combine them with extrinsic traits (e.g. geographic location, risk group, presence of a given resistance mutation), seeking to extract strain groups of specific interest or requiring surveillance.

**Results:** We propose a new method for obtaining such groups, which we call phylotypes, from a phylogeny having taxa (strains) annotated with extrinsic traits. Phylotypes are subsets of taxa with close phylogenetic relationships and common trait values. The method combines ancestral trait reconstruction using parsimony, with combinatorial and numerical criteria measuring tree shape characteristics and the diversity and separation of the potential phylotypes. A shuffling procedure is used to assess the statistical significance of phylotypes. All algorithms have linear time complexity. This results in low computing times, typically a few minutes for the larger data sets with a number of shuffling steps. Two HIV-1 data sets are analyzed, one of which is large, containing >3000 strains of HIV-1 subtype C collected worldwide, where the method shows its ability to recover known clusters and transmission routes, and to detect new ones.

**Availability:** This method and companion tools are implemented in an interactive Web interface (www.phylotype.org), which provides a wide choice of graphical views and output formats, and allows for exploratory analyses of large data sets.

**Contact:** francois.chevenet@ird.fr, gascuel@lirmm.fr

**Supplementary information:** Supplementary data are available at *Bioinformatics* online.

## 1 INTRODUCTION

Phylogenetic tools are commonly used to study virus evolution (Grenfell *et al.*, 2004), trace the origin of epidemics (Keele *et al.*, 2006), establish the mode of transmission (Hué *et al.*, 2005), survey the apparition of drug resistances (Hué *et al.*, 2009) or determine virus origin in different body compartments (Lamers *et al.*, 2011). The process involves the construction of phylogenetic trees, their visualization (e.g. Zaslavsky *et al.*, 2008) and their interpretation. Ancestral character reconstruction methods aid the interpretation, as extrinsic traits and their evolution can be mapped on the tree. Parsimony has been one of the first approaches to reconstruct ancestral characters. This method is remarkably fast, does not require any model assumption and is implemented in several popular phylogenetic programs (e.g. MacClade, Maddison and Maddison, 2003). More sophisticated, model-based, maximum-likelihood (ML) and Bayesian methods were developed for reconstructing ancestral characters (e.g. SIMMAP, Bollback, 2006; Lagrange, Ree and Smith, 2008). These methods are generally accepted to be more accurate than parsimony and account for various sources of uncertainty. However, they are time-consuming with large data sets and require a realistic evolutionary model to be available for the trait being considered. This is not an easy step, even with standard traits such as geographic location or morphological characters.

Despite the number of methods available for the inference of ancestral traits, there is little development for the interpretation of trait-annotated phylogenies. Most of the programs display the reconstructed ancestral states but do not allow for tests on ancestry and taxon clustering. For example, MacClade reconstructs ancestral characters and maps them in the phylogeny, but the resulting annotated tree needs to be interpreted visually. Other ML and Bayesian programs, such as BEAST (Drummond *et al.*, 2012), simultaneously reconstruct ancestral states and the phylogeny, and allow for testing. However, because of this simultaneous reconstruction, they need to be re-launched entirely when new traits are analyzed, which, combined with their computational heaviness, renders it difficult in the exploratory stages to select relevant traits for the data at hand. Moreover, model-based programs are mostly intended to specific trait types (e.g. geographical location, Lemey *et al.*, 2009, or molecular characters with SIMMAP). Finally, most (if not all) of ML and Bayesian programs are not able to deal with the huge amount of virus sequences available today, with data sets commonly comprising several thousand strains.

There is a need for a fast easy-to-use exploratory tool that can use phylogenies constructed with any of the most popular methods, while providing fast inference of ancestral traits and enabling hypothesis testing and visual data interpretation of evolutionary scenarios. In this article, we use and formally define, the concept of “viral phylotype”. Commonly, a phylotype is a biological type that classifies an organism by its phylogenetic relationship to other organisms. The term phylotype is taxon-neutral, thus one can choose the phylogenetic level at which the phylotype is described, depending on the question being examined. The term is commonly used in microbiology, and several tools have been developed to infer bacteria phylotypes (e.g. RAMI, Pommier *et al.*, 2009) or to study them from an ecological perspective (e.g. Picante, Kembel *et al.*, 2010, for analyzing the phylogenetic and trait diversity of ecological communities). Here we adopt a more specific virus view, combining (ancestral) traits with combinatorial and numerical criteria measuring tree shape characteristics and the diversity and separation of the phylotypes. Traits of extant and ancestral nodes are used to assess the relevance of phylotypes for the question being addressed. Numerical criteria are analogous to those used to define and study bacteria phylotypes. This method is implemented in “PhyloType”, a user-friendly Web interface that uses parsimony to reconstruct ancestral traits and a number of criteria to select phylotypes. A shuffling procedure is used to assess the significance of this selection process. The method is fast, allowing for the analysis of phylogenies comprising several thousands of taxa (strains). In the following, we describe the method and the Web interface, then show its application on two data sets, dealing with the epidemiological history of HIV-1 subtype A (HIV-1A) in Albania (Salemi *et al.,* 2008), and the global epidemic of HIV-1 subtype C (HIV-1C) using a large data set comprising ∼3000 strains collected worldwide (Jung *et al.*, 2012).

## 2 METHODS, CRITERIA AND ALGORITHMS

### 2.1 Phylotype definition and interpretation, method outline

A phylotype is a subset of studied taxa (strains) that share a common history. This history is 2-fold:

The first component is a phylogeny *T* of all taxa studied, which is one of the inputs of the method. The quality and relevance of results will, of course, depend on the accuracy of *T*, but *T* is not questioned in the analyses. *T* must be rooted and, again, the correctness of root location is essential. *T* may or may not be equipped with branch supports (e.g. bootstrap). More relevant results are obtained when branch supports are available and only well-supported parts of *T* are used. Clades (rooted subtrees) are the standard way to define subsets of taxa with common history from a rooted phylogeny. Here, a phylotype must be included in a clade of *T*, and the root of this clade must be the most recent common ancestor (MRCA) of the members of the phylotype. This MRCA is also called the root of the phylotype. In some cases (e.g. when studying the geographical origin of an epidemic), the common clade will be the entire phylogeny *T*. The common clade and MRCA property define the phylogenetic component of the common history of phylotypes.

The second component of this common history is induced by a set of traits or annotations attached to each of the taxa. For example, these annotations may describe the country of origin, the presence of a given resistance mutation, the risk group, the mode of transmission and so forth. These annotations are provided by the user and are the second input of the method. They are called primary annotations and can be combined in the Web interface to obtain, for example, secondary annotations representing regions of the globe by making a logical ‘OR’ of several countries. Different sets and combinations of annotations can be quickly explored, thanks to the speed of the method. Annotations used in a single analysis are mutually exclusive, but they may not be exhaustive (i.e. each taxon has at most one annotation).

**Fig. 1.**
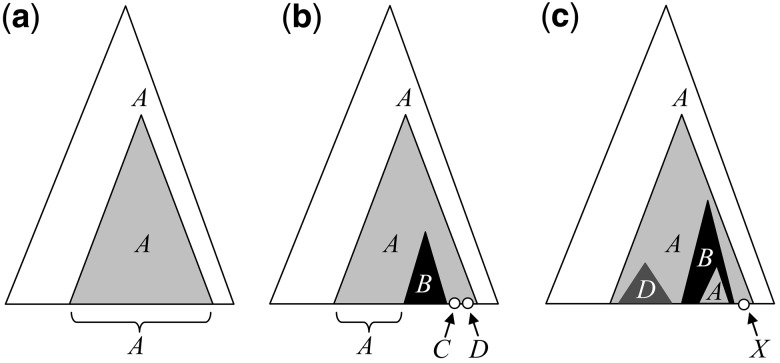
Three examples of phylotypes, ranked by increasing complexity

Beyond phylogeny, the members of a given phylotype share the same evolutionary history regarding the annotations being analyzed. The phylotype root must have a unique annotation, say *A*, which lasted until extant phylotype members that are annotated by *A*. Formally, within a clade with root *r*, every taxon *x* sharing the same annotation *A* as *r*, and for which *A* is conserved along the path from *r* to *x*, belongs to the phylotype defined by *r* and *A* (assuming the MRCA condition is fulfilled). As ancestral annotations are unknown, some inference method must be used. The PhyloType software currently uses parsimony, but other approaches are possible. Figure 1 presents examples to illustrate the phylotype concept:

- Figure 1a displays a phylotype that is as simple as possible. All taxa of the clade are marked by annotation *A* as well as the clade root and all intermediate nodes. Such a phylotype typically corresponds to the appearance of a new strain in a given country.
- Figure 1b shows an “intermediary” *A* phylotype, included in a clade that comprises taxa not annotated with *A*. Some of these non-*A* taxa belong to a (simple) *B* phylotype. Some others seem to be randomly mixed with the members of the *A* phylotype. Such a phylotype is typically seen with epidemics that start to break out from their country of origin (*A*).
- In Figure 1c, we see a “complex” or “ancient” phylotype. The original annotation, *A,* has become rare. The phylotype clade contains several nested clades defining sub-phylotypes, one of which annotated with *A*. Such a phylotype may correspond to ancient epidemics that have widely diffused among non-*A* taxa and have returned back to *A*.

Phylotypes may thus be simple clades (Fig. 1a), but they may also form hierarchical chains showing the succession of founder events (Fig. 1b and c). The approach differs from that commonly used for bacteria (e.g. RAMI, Pommier *et al.*, 2009; see also Zaslavsky *et al.*, 2008, for application to viruses), where clades with low genetic diversity are aggregated, producing clusters similar to our simple phylotypes. Here, a phylotype is a (potential) founder event associated with a unique annotation, for example, a country. Consider two phylotypes 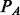 and 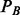, annotated with *A* and *B* respectively, with 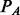 being the origin of 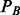 as shown in Figure 1b and 1c. This means that a strain (not necessarily originating from *A*) started to spread in *A* and produced a number of variants, one of which went to *B*, resulting in a new founder event 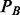. 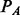 may be the origin of several founder events in *B*. The link between 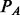 and 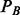 may be direct (*A* or *B* belong to the annotation set of every node in the path from 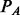 to 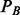 roots) or indirect (some nodes contains annotations that all differ from *A* and *B*).

The method to extract phylotypes can be summarized as follows (details are provided in the following sub-sections):

- Inference of ancestral annotations using parsimony.
- This ancestral reconstruction induces a set of potential phylotypes defined by the MRCA and path-conservation conditions (see above).
- Combinatorial, numerical and statistical criteria are used to select a set of relevant phylotypes from among all potential phylotypes.

### 2.2 Reconstruction of ancestral annotations

The PhyloType software uses parsimony to reconstruct ancestral annotations. To deal with incomplete information, we assume that non-annotated taxa are associated with the set of all possible annotations; other (informed) taxa must have a unique annotation. Annotations are treated as non-ordered discrete characters. Moreover, we assume that annotation changes are not oriented and thus use Fitch parsimony. Two standard options are available: ACCTRAN and DELTRAN (Swofford and Maddison, 1987). Both perform two tree traversals associating each tree node with the set of most parsimonious annotations, and requiring 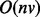 computing time to analyze a tree with *n* taxa and *v* annotations. The first bottom-up tree traversal is common to ACCTRAN and DELTRAN; it recursively computes the most parsimonious annotation sets associated to tree nodes with respect to their descendants. ACCTRAN and DELTRAN differ in the second top-down tree traversal to account for the three neighboring subtrees of a given node and resolve ambiguities: ACCTRAN chooses reversals over parallelisms when the choice is equally parsimonious, while DELTRAN does the opposite and favors parallelisms. ACCTRAN is typically used with morphological characters, while DELTRAN is often used with geographic annotations (e.g. Wallace *et al.*, 2007). Both procedures are based on extreme opposite choices to solve ambiguities, and it is a good practice to run both and compare the results to check that they are (nearly) identical. Note, however, that both ACCTRAN and DELTRAN may be unable to solve some ambiguities; the corresponding nodes then have “multiple” annotations. Moreover, the accuracy and relevance of ancestral annotations (and thus of the whole analysis) may depend on the representativity of the sample of sequences; if certain annotations are clearly over-represented, then parsimony (and any ancestral reconstruction method) will tend to over-predict those annotations, thus producing erroneous results. This phenomenon may become significant if, in addition, the tree contains numerous phylogenetic errors that spread over-represented annotations throughout the tree.

The potential phylotypes are defined by the clades having a unique annotation at their root. Phylotype members are obtained by following the paths where this annotation is conserved and unique, and the MRCA condition is accounted for by using the *Persistence* criterion (see below).

### 2.3 Combinatorial and numerical criteria

Combinatorial and numerical criteria are used to measure the relevance of potential phylotypes. A number of criteria are available in the Web interface. The choice of criteria and selection thresholds is left to users, who will test them interactively until a consistent and statistically supported picture is obtained. However, some minimal constraints (see below) are imposed to avoid meaningless analyses. All criteria are defined recursively and globally computed for all potential phylotypes in O(*n*) time (see Supplementary Material for formal definitions and algorithms).

#### Size

This criterion simply counts the number of taxa (members) in the potential phylotypes. Because having a phylotype containing a single taxon is meaningless, we impose that *Size* ≥2 is always present among phylotype selection criteria. Moreover, it is possible to express that some taxa correspond to several strains. We then count the number of strains in the potential phylotypes, instead of the number of taxa (tree leaves).

#### Different

This criterion measures the number of exceptions in the potential phylotypes. Let *P* be a potential phylotype defined by clade *C*; *Different* counts the number of sub-clades of *C* with annotations differing from *P*’s annotation. When such a sub-clade is found, it is counted as one, regardless of the number of covered taxa. For example, *Different* is equal to 3 for both main *A* phylotypes in Figure 1b and 1c.

#### Size/Different

*Size* and *Different* tend to be correlated. We expect large values of *Size* to be associated to relatively large values of *Different*. To account for both criteria simultaneously, we use their *Size/Different* ratio. Because having a phylotype containing many taxa and sub-clades with different annotations is meaningless, we impose that *Size/Different* with threshold ≥0.5 is always present among phylotype selection criteria. Default selection threshold is ≥1.

#### Total

This criterion counts the total number of taxa (strains) in the clade defining a potential phylotype, disregarding annotations. This criterion combines *Size* (to be maximized) and *Different* (to be minimized). To avoid conflicts with these two criteria, *Total* cannot be retained to select phylotypes, but its value may be of interest and is displayed by the software.

#### Persistence

This criterion measures the extent to which the root annotation *A* of the phylotype is conserved in its descendants. It is equal to the minimum depth where *A* is conserved, among all lineages starting from phylotype root. *Persistence* is used to prevent a phylotype from having a direct root descendant with annotation different from that of the root. It is easily seen that requiring *Persistence* to be >1 implies that the MRCA condition is fulfilled (both direct descendants have *A*, and thus both direct sub-clades contain taxa annotated with *A*). This ≥1 constraint is imposed in the interface, but larger thresholds can be chosen.

#### Local separation

We expect phylotypes to be well separated from the remaining taxa by a significant number of shared mutations. *Local separation* criterion is intended for this purpose and is equal to the length (i.e. expected number of substitutions per site) of the stemming branch of the phylotype. This criterion is available in community phylogeny software packages (e.g. RAMI, denoted as 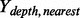).

#### Global separation

**Fig. 2.**
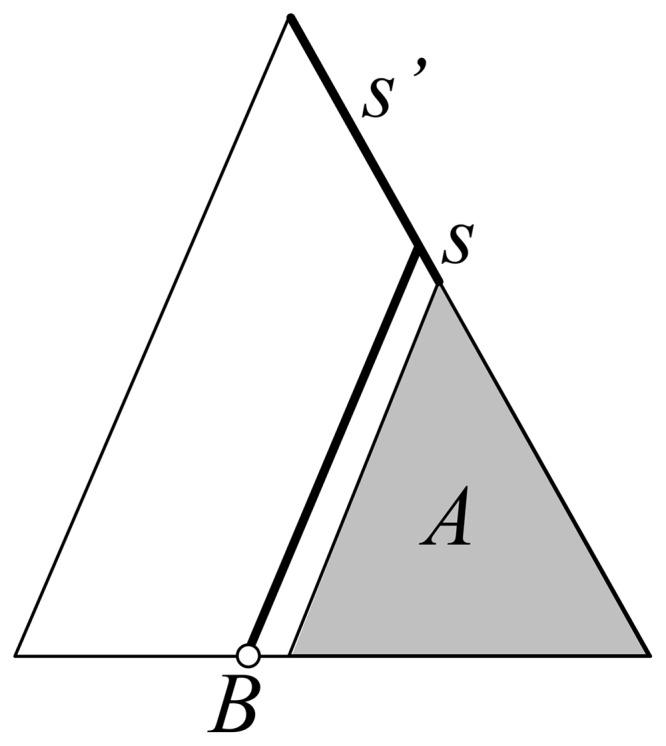
*Global separation* and *support*. The respective sizes of *A*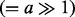 and *B* (=1) are used to compute global criterion values (see text and Supplementary Material for detailed formula and algorithm)

For a number of reasons (e.g. reconstruction errors), the stemming branch of a potential phylotype may be short, while the other branches separating the phylotype and phylogeny root are long and indicate a high separation of the phylotype from the rest of the tree. For example, in Figure 2, we see a large *A* phylotype with short stemming branch, but its sister group contains a single taxon with annotation *B*, and the clade joining both has a long stemming branch that should be accounted for when measuring the separation of the *A* phylotype. *Global separation* accounts for the lengths of all branches separating the phylotype and phylogeny root, but also incorporates the number of taxa contained in the sister clades encountered along the path. With our example of Figure 2, *Global separation* of the *A* phylotype is equal to *s* (local separation of *A*) plus *s*’ (local separation of *A*’s father) times 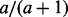, where *a* is the number of taxa in *A*, that is, nearly equal to 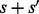. When the clade with *B* contains a large number of taxa, *Global separation* of the *A* phylotype is nearly equal to *s*.

This criterion is related to the 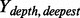 index provided by RAMI in that it counts all branches separating the phylotype from the tree root; however, 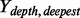 simply measures the total path length and does not account for sister group sizes. *Global separation* is also related to average distance criteria, for example, mean pairwise distance (MPD) in Picante, equal to the average distance between any taxon in the studied cluster and any taxon outside this cluster. An advantage of *Global separation* is that it requires linear computing times, while using average distances between taxon pairs requires (at least) quadratic computing times.

#### Diversity

This criterion measures the genetic diversity of phylotype members. It is equal to the average path-length distance between the members of the phylotype and the phylotype root. This criterion closely resembles the standard average genetic distance within the studied set of taxa (e.g. within-cluster MPD, used Picante), but avoid quadratic-time computations. It is close to Faith’s (1992) phylogenetic diversity (e.g. implemented in RAMI and Picante), but does not depend on the number of taxa.

#### Local and global separation/diversity

Separation and diversity indices depend on the tree scale. For trees with long branches, these indices will both tend to be high, and vice versa. To combine both aspects and avoid this scale effect, PhyloType provides users with these ratios: *Local separation/Diversity* and *Global separation/Diversity*.

#### Support

Any branch support (e.g. bootstrap, Bayesian posterior, aLRT) can be used to select phylotypes using a user-supplied threshold.

#### Global support

Just as with separation, *Support* may give a too local view. For example, in Figure 2, assume that *s* and *s*’ refer to branch supports, then the large *A* phylotype is well supported, even if *s* is low. PhyloType thus uses *Global support*, which is defined recursively as *Global separation*, but turning the summation of branch lengths into a maximum of branch supports to keep *Global support* less than the largest possible support value (e.g. number of bootstrap replicates). In Figure 2, *Global support* for the *A* phylotype is equal to 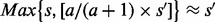.

### 2.4 Selection of most general phylotypes

Once all potential phylotypes have been evaluated using the criteria and thresholds defined by the user, we perform a top-down tree traversal to select the most general phylotypes satisfying all criteria. If an *A* phylotype satisfying all criteria is included in another *A* phylotype satisfying all criteria, then only the most general one will be selected, unless the path between the two contains one (or more) node(s) not annotated with *A*; in that case, both *A* phylotypes are selected. For example, in Figure 1c, assuming that both *A* phylotypes satisfy all criteria, then both are selected. In terms of infections and countries, this corresponds to an infection starting in *A*, diffused in *B* and then returning in *A*. Similarly, we compute whether the origin of phylotypes is direct or indirect (see above and Fig. 4 for examples). The corresponding *Search* algorithm runs in *O*(*n*) time and is provided in Supplementary Material. Lastly, when all phylotypes have been selected, we compute their values for all non-selection criteria. These values are displayed by the Web interface to help users to modify the selection criteria and analyze the results.

### 2.5 Assessing phylotype significance using shuffling

Once a set of phylotypes has been selected from the data, an essential question is whether or not these phylotypes have some statistical significance and clearly depart from a random selection of taxon subsets within the input phylogeny. For this purpose, we use a shuffling procedure, a common statistical tool that is used for similar purposes in phylogenetic software packages (e.g. MacClade) and to study the phylogeography of virus epidemics (e.g. Wallace *et al.*, 2007). Here, we randomly shuffle leaf annotations and proceed with phylotype selection using the same procedure and selection criteria used for the original data. This shuffling procedure is repeated a number of times, typically 100 or 1000, and *P*-values are computed. The implicit null hypothesis is that the annotations are randomly associated with the leaves of the tree. The *P*-value corresponds to the fraction of shuffled data sets in which one finds a phylotype with the annotation being evaluated and at least as large a criterion value as the observed phylotype. For example, in Table 1, we see the analysis performed with the HIV-1A, Albania data set (see below). One thousand shuffles were performed. The first Africa phylotype with *Size* (Sz) 44 has a *P*-value equal to 0/1000 regarding this criterion, meaning that among the 1000 random shuffles, none has been found with Africa annotation and *Size* ≥44; therefore, this phylotype is highly significant regarding the *Size* criterion. However, it is not significant regarding the *Persistence* criterion; the *P*-value is equal to 867/1000 meaning that 867 shuffled data sets were found, with Africa annotation, all selection criteria (Table 1) and *Persistence* ≥2. Globally this phylotype is statistically significant, as it is unlikely that such a large phylotype can be found by chance. The second Albania phylotype is statistically significant (*P*-value ≤1%) for the three selection criteria.

**Table 1.** Detailed table of the significant phylotypes found with HIV-1A, Albania data set

Pi	Anc	A	Cov (%)	**Sz**	***P*s**	**Sz/Df**	Tt	Df	Sl	Sg	Dv	Sl/Dv	Sg/Dv	**Sp**	Spg	AnB
1	root	Africa	88	**44**	**2**	**2.750**	152	16	0.009	0.009	0.172	0.051	0.051	**1.000**	1.000	–
				***0/1000***	***867/1000***	***201/1000***										
17	14	Albania	97	**30**	**3**	**30.000**	32	1	0.017	0.059	0.024	0.718	2.415	**0.960**	0.960	–
				***0/1000***	***11/1000***	***1/1000***										
251	1	EastEurope	80	**8**	**1**	**8.000**	9	1	0.057	0.065	0.035	1.646	1.886	**1.000**	1.000	–
				***0/1000***	**0*/1000***	***0/1000***										
14	1	Greece	69	**27**	**2**	**13.500**	60	2	0.009	0.073	0.066	0.132	1.097	**0.880**	0.880	–
				***0/1000***	***424/1000***	***10/1000***										

Selection criteria (displayed with bold characters) are *Size* (Sz ≥5), *Persistence* (Ps ≥1), *Size/Different* (Sz/Df ≥1) and *Support* (Sp ≥70%). *P*-values (in italic) are given as fractions, where the denominator indicates the number of shuffles. The analysis was run with ACCTRAN option. Pi, identifier of phylotype root; Anc, phylotype origin; A, annotation; Cov (%), coverage, i.e. percentage of taxa annotated with A that belongs to the phylotype; Tt, *Total*; Sl, *Local separation*; Sg, *Global separation*; Dv, *Diversity*; Spg, *Global support*; AnB, list of ‘Breaking’ annotations when the origin is indirect (see text and examples in Fig. 4).

*Size* is the most discriminating criterion, and the use of the shuffling procedure is especially relevant with this criterion. Assume that some annotation is highly represented in the data set; then we expect relatively large phylotypes having this annotation to be found by chance. On the other hand, rare annotations have a low probability of giving rise to phylotypes by chance. The shuffling procedure quantifies this random effect and can be used with a user-supplied significance level to discard phylotypes that could be found by chance due to the abundance of certain annotations. As multiple testing is performed, a low significance level (e.g. 1%) is recommended. Note, however, that sample size plays a crucial role. With limited sampling, low-sized non-significant phylotypes are to be expected. With additional samples, some of these phylotypes will become significant and the others will be discarded.

To obtain reliable *P*-value estimations, the number of shuffles has to be large enough; for example, having a *P*-value = 0/10 (i.e. with 10 shuffles) does not mean that the *P*-value is equal to 0. This is why we prefer not to provide the *P*-value as a percentage. Thanks to the speed of our selection algorithms, performing shuffling with 1000 iterations still requires low computing times, for example, ∼12 min for the current version of the Web interface (powered by Intel Xeon X5650 CPUs, without any parallel implementation) with our large HIV-1C data set comprising ∼3000 taxa and 14 annotation values.

### 2.6 PhyloType Web interface

The Web interface (www.phylotype.org) divides PhyloType analyses into the five steps summarized below. All required details are provided in the downloadable User Guide. The Web site includes data set examples showing the input formats and expected output files.

#### Input

This step enables to copy/paste or upload a phylogenetic tree and its annotations. The tree is in Newick format with branch lengths and branch supports (optional). Annotations are in CSV (Comma-Separated Values) format, possibly with missing values. A third, optional, input is the number of strains attached to each taxon (default is 1, see 2.3).

#### Tree

This step is optional. Several methods (e.g. midpoint, outgroup) are available to root the tree, when the user tree is unrooted. The *Tree* step enables a visualization of the tree with the posting of branch lengths and supports.

#### Annotation

This step is also optional. The interface displays the annotation variables with all their possible values and respective frequencies among taxa. The *Annotation* step allows for logical combinations of annotations. For instance, an ‘OR’ connector allows for the aggregation of values (e.g. making global ‘regions’ from ‘countries’ annotations). Other operators are available, such as logical ‘AND’, duplication, deletion, etc. These combinations of annotations can be downloaded for further analyses.

#### Analysis

This step corresponds to the PhyloType analysis itself. The user selects the criteria to be used for identifying phylotypes. Twelve criteria are available (see 2.3). Each criterion has a default threshold, which can be modified to be more or less stringent. An annotation variable must be selected, and, for this variable, a set of annotations to study is chosen. The parsimony option (ACCTRAN or DELTRAN) is selected, as well as the inclusion (or not) of the outgroup in the analysis. Lastly, the user can decide to perform shuffling, define the number of iterations and provide a *P*-value for phylotype selection using the *Size* criterion. PhyloType results are first summarized in an overview table that displays the number of phylotypes for each annotation value and various statistics (Supplementary Table S3). PhyloType analyses are fast, and it is easy to tune the parameter settings to explore the data with respect to this overview table.

#### Output

**Fig. 3.**
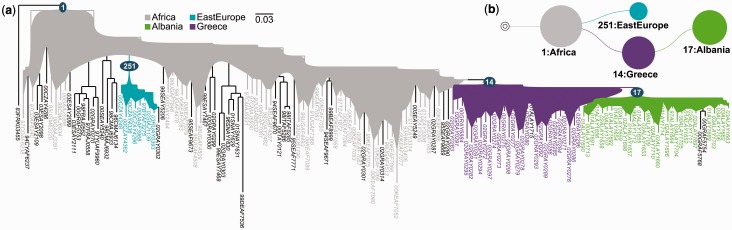
Tree graphics obtained in the study of the epidemiological history of HIV-1A in Albania (Salemi *et al.*, 2008). (**a**) Phylogenetic tree in “background” format: selected phylotypes and their strains are colored; colored regions comprise all (uniquely annotated) nodes on the path from the phylotype root to the phylotype members; not colored (black) strains do not belong to any phylotype; the root node identifiers of phylotypes are provided, to be used in conjunction with the detailed table (Table 1). (**b**) Phylotype map, summarizing the information contained in phylogenetic tree (a); circle surface is proportional to the *Size* value (number of members) of the phylotype

**Fig. 4.**
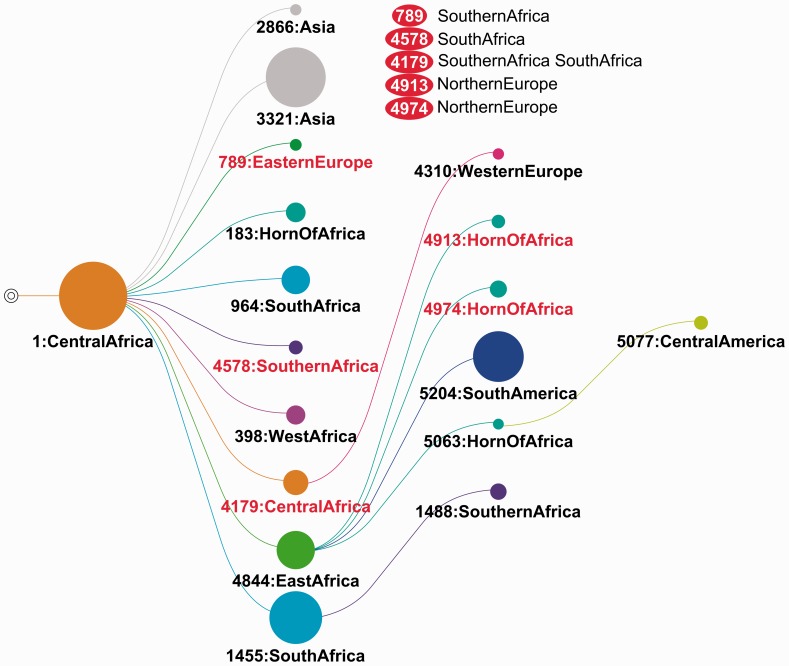
Phylotype map (ACCTRAN) of the worldwide study of HIV-1C. Some of the phylotypes (colored in red) have indirect origin; for example, 789: Eastern Europe, with Southern Africa annotation(s) along the path to 1: Central Africa

This final step gives access to the detailed results of PhyloType. A first table (Table 1) lists all selected phylotypes with corresponding statistics and taxa. Graphical outputs are available, such as phylogenies with color-encoded phylotypes or phylotype maps (Figs 3 and 4). All the input/output are available for download: original and rooted trees with node identifiers; annotations (primary and combined); overview and detailed phylotype tables; ready-to-print trees and map graphics in various formats.

#### Implementation

The PhyloType pipeline is based on Tcl/Tk CGI scripts. PhyloType will be available soon as a free stand-alone package for Windows and Unix-like systems, including OSX. It will then be possible to integrate this tool in diverse environments and virus database resources.

## 3 RESULTS WITH TWO HIV-1 DATA SETS

In this section, we show the application of PhyloType software on two HIV-1 data sets, the first one (HIV-1 subtype A) with limited size to show the outputs and advantages of the method, the second one (HIV-1 subtype C) to demonstrate its efficiency to deal with large numbers of taxa and annotations. All input data and PhyloType results are available from www.phylotype.org/SupMat/.

### 3.1 Epidemiological history of HIV-1A in Albania

In this case, we applied PhyloType to a relatively small data set of *pol* sequences from 152 HIV-1 subtype A strains. This data set, the sequences and their alignment were derived from the study by Salemi *et al.* (2008) on the origin of subtype A in Albania, where this subtype is widespread, while the rest of Europe is mostly affected by subtype B. The sequences used in this study are from Africa (50) and Europe (102), with 31 sequences collected in Albania and 39 in Greece, the suspected origin of the Albanian epidemic. The isolate HXB2 (HIV-1B) was used as the outgroup. As in Salemi *et al.* (2008), the phylogeny was inferred by ML. We used PhyML (Guindon *et al.*, 2010) with GTR+I+Γ4 (following jModelTest, Posada, 2008), the SPR option to search the tree topology and aLRT SH-like branch supports.

The primary annotations in this data set are the countries in which the studied sequences were collected. These annotations were grouped into five geographic zones (as in Fig. 1a of Salemi *et al.*, 2008): Africa, Western Europe, Eastern Europe, Greece and Albania (see Supplementary Table S1 for details). The criteria chosen for the PhyloType analysis were *Size* ≥5, *Persistence* ≥1, *Size*/*Different* ≥1 and *Support* ≥0.7. The ACCTRAN parsimony method was selected and 1000 shuffles were performed to test phylotype significance. Only those phylotypes whose *P*-value for *Size* is ≤1% were retained. These options, selection criteria and thresholds correspond to PhyloType’s default parameter settings.

Results are provided in Table 1 and Figure 3, which shows two types of graphical outputs provided by PhyloType: (a) a phylogeny in which the selected phylotypes are represented by colored regions; (b) a phylotype map that indicates the succession of founder and migratory events. DELTRAN results are nearly identical, the only difference (Supplementary Fig. S1) is that the large African phylotype includes six additional sequences due to the removal of some ancestral ambiguities. Moreover, we checked the robustness of the results regarding selection parameters. We observed high stability (see Supplementary Fig. S2 for details), except when (as expected) we used a low *Size* threshold of 3 and did not perform any shuffling, which resulted in artifactual phylotypes. This illustrates the interest of the shuffling procedure, which should be used in all analyses to discard non-significant phylotypes.

Four phylotypes are found (Table 1) covering 72% of the sequences: an ancestral African phylotype (containing 88% of African strains); a Greek phylotype, clearly supported, with most of Greek strains (69%); a small East European phylotype [8 strains out of 10; 4 (out of 5) are Czech]; an Albanian phylotype with Greek origin containing almost all Albanian strains (97%), with strong aLRT support (0.96) and high significance. PhyloType analysis thus reaches the same main conclusions as Salemi *et al.* (2008): ‘The finding that 99% of the HIV-1A Albanian sequences could be traced back to a unique MRCA suggests a single major introduction of HIV-1A from Greece followed by local epidemic spread’. One can also see the importance of allowing exceptions in these analyses, as the large Greek phylotype includes one Albanian sequence (outside the Albanian phylotype), whereas the Albanian phylotype includes two Greek sequences. As noted by Salemi *et al.* (2008), ‘This could indicate a limited ongoing viral gene flow between the two neighboring countries’. One can also observe multiple introductions of HIV-1A in Europe, including in Greece. Lastly, the analysis reveals the small East European phylotype, not pointed out by Salemi *et al.* (2008), but which is highly significant (Table 1). This phylotype contains most of the East European strains, plus one Greek exception, and has aLRT support of 1.0. It could correspond to a founder event followed by restricted spreading in Eastern Bloc countries, before its transformation. Additional studies and sequences would be necessary to confirm this hypothesis. However, we see here the advantage of a fast method such as PhyloType, which makes it possible to reveal and quantify (objective selection criteria, statistical significance using shuffling) the presence of clusters of interest, a task that is difficult to achieve using visual inspection, even on such a small data set.

### 3.2 Worldwide evolutionary history of HIV-1C

To demonstrate the abilities and efficiency of PhyloType, we applied the software to a large amount of data related to the HIV-1 subtype C epidemic on a worldwide scale. This subtype is the cause of nearly half of all HIV-1 infections. It is highly prevalent in southern and eastern (mainly Burundi and Ethiopia) Africa, along with irregular distribution throughout the rest of the world, including some highly contaminated countries such as India (Hemelaar *et al.*, 2011). We used the *pol* sequences of (Jung *et al.*, 2012), corresponding to all subtype C *pol* data available in the Los Alamos HIV database at the time of this study, to which 18 sequences from Senegal were added. We removed 45 sequences indicated as recombinant using the last version of SCUEAL (Kosakovsky Pond *et al.*, 2009), thus obtaining a set of 3036 ingroup sequences collected in 60 countries (Supplementary Table S2). Moreover, to root the tree, we added 35 reference outgroup sequences extracted from the Los Alamos HIV database (non-C, subtype A to K). The phylogeny was constructed using PhyML with GTR+I+Γ4, SPR option and aLRT SH-like branch supports. All alignment sites were used to build this tree, including sites associated to drug resistances, as their deletion showed little impact (Jung *et al.*, 2012; see this article for details on data, alignment and phylogeny calculation).

As with the previous study, we grouped countries in major areas to have enough strains for each group and to be able to extract synthetic and significant information. A total of 14 groups were used (see Supplementary Table S2 for details and Supplementary Figs S4 and S5 for other groupings and corresponding results): Central Africa [644 strains, mostly from Zambia, plus a few strains collected in the Democratic Republic of Congo (DRC) near the Zambian border]; South Africa (including Swaziland, 731 strains); Southern Africa (other southern Africa countries, 328 strains); East Africa (188 strains, mostly from Burundi and Tanzania); Horn of Africa (118 strains, mostly from Ethiopia); West Africa (63 strains, mostly from Senegal); Asia (366, mostly from India); South America (245, mostly from Brazil); Central America (26, mostly from Cuba); North America (9, all from the USA); Northern (106), Western (67), Southern (92) and Eastern (53) Europe.

The selection criteria for PhyloType were *Size* ≥10, *Persistence* ≥1, *Size*/*Different* ≥1 and *Support* ≥0.7, that is, the default parameter setting, except for *Size* (10 instead of 5) because of the large sample used here. The influence of these parameters is studied in the Supplementary Material and discussed below. We used both ACCTRAN and DELTRAN with 1000 shuffles and a significance level for the *Size* criterion of 1%. The results with ACCTRAN are reported in Figure 4, Supplementary Tables S3 and S4. Eighteen phylotypes are found covering 58% of the sequences (results with DELTRAN are similar, see Supplementary Fig. S3 and further discussion):

#### Epicenter in Central Africa

The analysis suggests (phylotype no. 1, coverage of 69%) that the epicenter of the HIV-1C epidemic is located in southern Central Africa. This annotation includes strains collected in Zambia and DRC. The latter was previously identified as being the epicenter of viruses belonging to the pandemic group M of HIV-1 (Vidal *et al.*, 2000). Nonetheless, the root of this phylotype does not correspond to the root of the phylogeny, and it is possible that the origin of HIV-1C is located in a different region, just as is the case for HIV-1M itself (Keele *et al.*, 2006).

#### Dissemination around the African continent

The virus spread from Central Africa across the entire African continent, that is, directly into Southern African countries (phylotype no. 4578), South Africa (nos. 964 and 1455), East Africa (no. 4844), West Africa (no. 398) and Horn of Africa (no. 183), or indirectly to the Horn of Africa (nos. 4913, 4974 and 5063) and Southern African countries (no. 1488), passing through East and South Africa, respectively. The direct filiation from East Africa to Horn of Africa is doubtful for two phylotypes (nos. 4913, 4974) with ACCTRAN, as the links are broken by Northern Europe annotations (Fig. 4), but this finding is not observed with DELTRAN (Supplementary Fig. S3), which infers a direct origin in both cases. Even if they differ slightly, the two parsimony options establish a dual geographical (Central and East Africa) origin of the Horn of Africa epidemic, which could be a plausible explanation for the observation of the C and C’ sub-clusters in Ethiopia (Abebe *et al.*, 2000). Thomson and Fernández-García (2011) demonstrated that the majority of East and Horn of Africa strains are grouped within one clade, as our analysis suggests once again (phylotype no. 4844), with the exception of the small Horn of Africa phylotype no. 183 (directly descending from Central Africa). With ACCTRAN, PhyloType finds a Central Africa phylotype (no. 4179) that is a descendant of the major Central Africa phylotype (no. 1); the origin is indirect, possibly indicating a symmetrical flow and a return of the epidemic to the center from southern regions, but DELTRAN does not confirm this finding and groups the corresponding strains into a single large ancestral Central Africa phylotype (coverage of 86%). Lastly, we reach the prediction made in (Jung *et al.*, 2012) on a possible Zambian origin of the HIV-1C epidemic in Senegal (WestAfrica phylotype no. 398), especially in the MSM population. However, the African phylotypes altogether cover only ∼50% of the African strains: some regions are well-covered (>70%, Central Africa, East Africa, Horn of Africa), some are in between (∼50%, South and West Africa), while the Southern African countries (Botswana, Mozambique, Malawi…) have a low coverage (∼12%), likely due to their passing position between Central Africa and South Africa, with numerous introductions and bi-directional exchanges.

#### From Central Africa to Asia

Our analysis indicates that the two Asian phylotypes (nos. 2866 and 3321, total coverage of 96%) originated in southern Central Africa (phylotype no. 1), which differs from the origin predicted for India (97% of Asian strains) by (Shen *et al.*, 2011), suggesting South Africa instead. However, this study used few strains from Zambia (<50) and was based on the *env* gene. Moreover, a recent study performed on the three genes (*gag*, *pol* and *env*) was not able to determine the origin of the Indian cluster in any of those genes (Neogi *et al.*, 2012). Their analysis, like our own, suggests that the HIV-1C epidemic in India was subject to multiple introductions with a major cluster.

#### Multiple introductions into Europe

The Western Europe phylotype (no. 4310, descendant of Central Africa) only includes strains originating from Belgium, a country with historical and economic links with DRC. The Eastern Europe phylotype (no. 789) also contains strains from Romania exclusively. However, a small fraction (∼33%) of the strains from Belgium and Romania are included in these two phylotypes, and most of the European strains (93%) are not included in any phylotype. This confirms multiple introductions and complex transmission chains of HIV-1C in Europe, as already pointed out in numerous studies.

#### From East Africa to South America

The analysis suggests that the HIV-1C epidemic propagated from East Africa (phylotype no. 4844) towards South America (no. 5204, coverage of 99%), a link already identified on several occasions (e.g. Véras *et al.*, 2011). Let us note that, with this data set containing few strains (3) collected in England, it is not possible to confirm or infirm the hypothesis that England played a key role in spreading HIV-1C in Brazil (de Oliveira *et al.*, 2010).

#### Origin of the epidemic in Cuba

The Central America phylotype (no. 5077, coverage of 65%) contains strains from Cuba exclusively, a country with numerous links to Africa. With ACCTRAN (Fig. 4), we find a transmission chain from East Africa to Horn of Africa and then Central America, while with DELTRAN (Supplementary Fig. S3) the intermediary Horn of Africa step is not found and the filiation from East Africa to Central America (i.e. Cuba) is direct. Note, however, that 35% of Central America strains are not covered by any phylotype, which suggest multiple introductions into Cuba and surrounding countries.

#### Discussion, influence of options and selection parameters

As ACCTRAN and DELTRAN are two heuristics performing extreme choices to solve ancestral annotation ambiguities, their comparison enables us to check the robustness of results regarding parsimony-based ancestral reconstructions. We have already indicated above certain differences between the two analyses. A general trend (with this data set and others) is that DELTRAN returns fewer ancestral ambiguities and fewer indirect origins than ACCTRAN (none versus five, Supplementary Fig. S3). As a consequence, DELTRAN covers more sequences than ACCTRAN (64% versus 58%) and finds one more phylotype (19 versus 18). Moreover, six small phylotypes (<150 sequences in total) are found by one method but not the other. However, both methods mostly agree. A number of phylotypes are identical (8) or nearly indentical (7 with similarity >80%) in both analyses, and the geographical history is the same for 88% of the covered sequences (see Supplementary Fig. S3 for more results and explanations on these measures). Overall, this comparison gives us high confidence in most of the results of the PhyloType analysis and puts some others into perspective (e.g. origin of the Cuba phylotype, see above).

A good practice is to try alternative annotation groupings and measure their influence on the results. Thus, we tested two other solutions, using the original country annotations and, on the opposite, grouping countries into continents (Europe, Asia and America) and subcontinents (Central Southern Africa, Other Africa). As expected, when using broad (sub)continent annotations (Supplementary Fig. S4), more sequences are covered (85%) but the phylogenetic map is oversimplified and not informative: eight original phylotypes (among 18) are recovered, but nine are included in two large African phylotypes that depict a coarse (but consistent) history of the epidemic in Africa, from Central and Southern countries to the rest of the continent. When using country annotations (Supplementary Fig. S5), the number of covered sequences remains analogous and the similarity with original analysis is high (89%), but we have more ancestral annotation ambiguities and are unable to establish some of the filiations and results found previously. For example, we do not find any phylotype related to DRC, and that the epicenter of the epidemic seems to be situated in the DRC-Zambia region. Altogether, these results indicate that the grouping step must be conducted with care using additional (geographical, historical…) knowledge and that an exploratory phase is recommended to evaluate the relevance of different annotation groupings.

Lastly, we checked the robustness of the results regarding selection parameter values. When lowering the *Size* threshold to 5 (instead of 10), the analysis remains highly similar (97%), but we find eight additional small phylotypes (49 sequences in total); on the opposite, when the *Size* threshold is equal to 20, the results are still consistent but seven phylotypes are lost (Supplementary Fig. S6). The *Size*/*Different* criterion is more difficult to tune. Basically, it represents the fraction of exceptions that one accepts in phylotypes. When too low, some phylotypes become meaningless and turn out to be non-significant using the shuffling procedure; when too high, some relevant phylotypes are lost. Here, the results do not change much in the 0.5, 2.0 range (Supplementary Fig. S7); 0.5 is a bit too low; values between 1.0 and 2.0 seem to be convenient and result in similar meaningful phylotypes. With high threshold values, large phylotypes are lost and broken into numerous small phylotypes.

## 4 CONCLUSION

To summarize results regarding the HIV-1 subtype C pandemic: PhyloType recovers a number of already-identified transmission chains (e.g. East Africa to Brazil), contradicts a few others (e.g. origin of Indian epidemic) and suggests some new routes (e.g. two different geographical origins of the Ethiopian epidemic). Note that PhyloType reveals potential founder events, rather than gene flows as proposed by Slatkin and Maddison (1989) and used in numerous studies (e.g. Wallace *et al.*, 2007; Véras *et al.*, 2011). PhyloType results are obtained rapidly, in a matter of minutes, through a user-friendly Web interface. Therefore, we believe that PhyloType software has the potential to be extremely useful for exploring and interpreting the large virus phylogenies, which are available today and should become the norm in the near future. The application’s speed should be a major asset in surveillance tasks. However, for many viruses (e.g. Hepatitis B), strain annotations (countries, dates…) are not yet as easily obtained from the databases as they are with HIV-1, which should imply the need for some significant preliminary work before launching PhyloType analyses. Moreover, representative samples are still lacking for several viruses (e.g. Nonovirus), and sampling efforts will have to be made to use PhyloType (and any other similar tool) effectively.

Several directions deserve further exploration, most notably the implementation of simple and computationally efficient probabilistic models, to be used instead of parsimony to infer ancestral annotations, and the automatic combination of annotation values, as performed by other methods (e.g. DIVA, Ronquist, 1997; Lagrange, Ree and Smith, 2008). Moreover, PhyloType could be used for radically different purposes, such as exploring large protein families associated with differentiated or specialized functions.

## Supplementary Material

Supplementary Data
