# Supplementary material for: Searching for virus phylotypes
Source: Bioinformatics. 2013 Jan 17;29(5):561–70. doi: 10.1093/bioinformatics/btt010 (PMC3582263; doi:10.1093/bioinformatics/btt010)
Supplement: Supplementary Data [file supp_29_5_561__index.html]

Searching for virus phylotypes — Searching for virus phylotypes — Supplementary Data 

# Searching for virus phylotypes

## Supplementary Data

files

**Files in this Data Supplement:**

- Supplementary Data - pdf file
