# Supplementary material for: Searching for virus phylotypes
Source: Bioinformatics. 2013 Jan 17;29(5):561–70. doi: 10.1093/bioinformatics/btt010 (PMC3582263; doi:10.1093/bioinformatics/btt010)
Supplement: Supplementary Data [file supp_btt010_SupMat_ChevenetEtAlGascuel_bioinformatics2012_R1.pdf]

SUPPLEMENTARY MATERIAL

Original Paper

Searching for Virus Phylotypes

François CHEVENET[1,2], Matthieu JUNG[1,3], Martine PEETERS[3], Tulio DE OLIVEIRA[4] and Olivier GASCUEL[*,1]

[1]Institut de Biologie Computationnelle, LIRMM, UMR 5506 CNRS – Université Montpellier 2, FRANCE

[2]MIVEGEC, CNRS 5290, IRD 224, Universités Montpellier 1 et 2, FRANCE

[3]TransVIHMI, UMI233, IRD – Université Montpellier 1, FRANCE

[4]Africa Centre for Health and Population Studies, University of KwaZulu-Natal, SOUTH AFRICA

* Corresponding author: gascuel@lirmm.fr

- **Criteria and selection algorithms** **pp. 2-5**

- **Sup. Table S1: Grouping of countries for the HIV-1A, Albania study** **pp. 6**
- **Sup. Table S2: Grouping of countries for the worldwide study of HIV-1C** **pp. 7**
- **Sup. Table S3: Summary table with ACCTRAN for the worldwide study of HIV-1C** **pp. 8**
- **Sup. Table S4: Detailed table with ACCTRAN for the worldwide study of HIV-1C** **pp. 9**

- **Sup. Figure S1: Background phylogeny with DELTRAN for the Albania study of HIV-1A** **pp. 10**
- **Sup. Figure S2: Stability of the results re. parameter values for the HIV-1A, Albania study** **pp. 11**
- **Sup. Figure S3: ACCTRAN/DELTRAN comparison for the worldwide HIV-1C study** **pp. 12**
- **Sup. Figure S4: Results using (sub)continent annotations for the worldwide HIV-1C study** **pp. 14**
- **Sup. Figure S5: Results using country annotations for the worldwide HIV-1C study** **pp. 16**
- **Sup. Figure S6: Stability of the results re. *Size* value for the worldwide HIV-1C study** **pp. 18**
- **Sup. Figure S7: Stability of the results re. *Size/Dif.* value for the worldwide HIV-1C study** **pp. 19**

© Oxford University Press 2005

Criteria and selection algorithms

***Size*:** This criterion counts the number of taxa (members) in the potential phylotypes. Let *P* be a potential phylotype, *L* be its left-descendant sub-clade, and *R* its right-descendant sub-clade (*i.e.* the clade defining *P* is composed of the two sub-clades *L* and *R*). Let *A* be the annotation of *P*. *Size* is calculated as follows:

$Size(P, A)$

If $Annotation(P) \neq A$, then 0

Elseif $P = leaf$, then $Number(P)$

Else $Size(L, A) + Size(R, A)$

where $Number(P)$ is the number of strains corresponding to taxon *P* (1 by default). To compute the *Size* value of all potential phylotypes in $O(n)$, where *n* is the number of taxa, we use an appropriate data structure to memorize the results, and perform a post-order tree traversal that computes the criterion value from the tree leaves to the root. The same strategy is used for other criteria to turn local recursive definitions into global computations over the whole tree and all potential phylotypes.

Different: This criterion counts the number of sub-clades included in potential phylotype *P*, which have different annotations of *P*'s. *Different* is calculated as follows (notation is the same as for *Size*, which holds for all criteria below):

$Different(P, A)$

If $Annotation(P) \neq A$, then 1

Elseif $P = leaf$, then 0

Else $Different(L, A) + Different(R, A)$

Total: This criterion simply counts the total number of taxa (strains) included in the potential phylotype:

$Total(P)$

If $P = leaf$, then $Number(P)$

Else $Total(L) + Total(R)$

Persistence: This criterion measures extent to which the root annotation *A* of the phylotype is conserved in its descendants. *Persistence* is recursively computed as follows, starting from the phylotype root and its annotation *A*:

$$Persistence(P, A)$$
If $(P = leaf)\ OR\ (Annotation(L) \neq A)\ OR\ (Annotation(R) \neq A)$,
Then 0,
Else $1 + Min\{Persistence(L, A), Persistence(R, A)\}$

Global separation: This criterion accounts for the lengths of all branches separating the phylotype and phylogeny roots. *Global separation* also incorporates the number of taxa (measured by *Total*) contained in the sister clades encountered along the path. *Global separation* is defined recursively and computed as follows:

$$Global\_separation(P)$$
If $(P \neq tree\ root)$,
Then $Local\_separation(P) + \dfrac{Total(P)}{Total(F)} Global\_separation(F)$,
Else 0.

F is the father node of *P*, and $P \neq tree\ root$ tests whether *P* is equal to the root of the input phylogeny. When the ingroup and outgroup are defined, we instead use the root of the ingroup to compute *Global separation* and stop the recursion. With our example of Figure 2, *Global separation* of *A* phylotype is equal to *s* (local separation of *A*) plus *s*' (local separation of *A*'s father) times $a/(a+1)$, where *a* is the number of taxa in *A*, that is, nearly equal to $s+s'$. Assume now that the clade with *B* contains a large number of taxa. In that case, the long branch *s*' has little weight, as corresponding mutations are shared by a number of non-*A* taxa, and then the global separation of the *A* phylotype is nearly equal to *s*.

Diversity: This criterion measures the genetic diversity of phylotype members. It is equal to the average path-length distance between the members of the phylotype and the phylotype root. *Diversity* is computed using:

$$Diversity(P)$$
$$Sum(P, Annotation(P)) / Size(P, Annotation(P)).$$

where $Sum(P, A)$ is recursively defined by (*l* and *r* are the lengths of the stemming branches of *L* and *R*, respectively):

$$Sum(P, A)$$
If $(Annotation(P) = A)\ AND\ (P \neq leaf)$
Then $Sum(L, A) + l \times Size(L, A) + Sum(R, A) + r \times Size(R, A)$
Else 0.

Global support: This criterion accounts for the support of all branches separating the phylotype and phylogeny root. Global support also incorporates the number of taxa (measured by *Total*) contained in the sister clades encountered along the path. Global support is defined as follows (*F* is the father node of *P*):

$Global\_support(P)$

If $(P \neq tree\ root)$,

Then $Max\left\{Support(P),\ \frac{Total(P)}{Total(F)} Global\_support(F)\right\}$,

Else 0.

Here we use a maximum, instead of a summation (used for *Global separation*, see above), to keep *Global support* less than the largest possible support value (e.g. number of bootstrap replicates, 1.0 with Bayesian and aLRT supports).

Selection of most general phylotypes: Once all potential phylotypes have been evaluated using the criteria and thresholds defined by the user, we perform a top-down tree traversal to select the most general phylotypes satisfying all criteria. If an *A* phylotype satisfying all criteria is included in another more general *A* phylotype satisfying all criteria, then only the most general one will be selected, unless the path between the two contains one (or several) non-*A* annotations; then both *A* phylotypes are selected. To check this condition, the selection algorithm uses a *Record* variable that memorizes the root annotation of the clade being explored. The algorithm is launched with the whole input phylogeny, $Record = \varnothing$, and is defined recursively by:

$Search(P, Record)$

If $P = leaf$, Then $stop\ Search$;

Elseif $Annotation(P)\ is\ unique\ AND\ Annotation(P) \neq Record\ AND\ AllCriteria(P)$

Then $select\ P\ and\ set\ Record = Annotation(P)$;

Elseif $Record \notin Annotation(P)$

Then $set\ Record = \varnothing$;

$Search(L, Record)\ and\ Search(R, Record)$.

When all phylotypes have been selected, we compute their values for all non-selection criteria. Overall the whole procedure can be summarized as follows:

Phylotyping (P, A)

Input a phylogeny P *with leaf (and ancestral) annotation set* A;

Compute ancestral annotations using parsimony (DELTRAN/ACCTRAN option);

Compute all selection criteria for all nodes of P *with unique annotation*;

Search $(P, \varnothing)$;

For all selected phylotypes, compute the values of remaining criteria;

Output the set of selected phylotypes with their criterion values.

All steps in this procedure require linear computing times in the number *n* of taxa (parsimony computations require $O(nv)$, where *v* is the number of annotation values). Less than 1 second is required to select phylotypes with our large data set comprising ~3,000 taxa and 14 annotation values; a shuffle with 1,000 steps is performed in ~720 seconds (Intel Xeon X5650).

Region	Strain number	Country (strain number)
**Africa**	50	CM, Cameroon (6); CD, Democratic Republic of Congo (2); GAB, Gabon (2); KE, Kenya (19); RW, Rwanda (1); SN, Senegal (3); SD, Sudan (2); TZ, Tanzania (4); UG, Uganda (11)
**East Europe**	10	BL, Bielorussia (1); CZ, Czech Republic (5); RU, Russia (1); SL, Slovenia (1); UA, Ukraine (1); YU, Former Yugoslavia (1)
**West Europe**	22	BE, Belgium (1); CY, Cyprus (1); FR, France (2); DE, Germany (1); IT, Italy (3); SP, Spain (6); SE, Sweden (8)
**Albania**	31	Alb, Albania (31)
**Greece**	39	GR, Greece (39)

Supplementary Table S1. Grouping of countries into regions and continents, for the 152 strains of the HIV-1A, Albania study (Salemi *et al*., 2008).

Region	Number of strains	Country (number of strains)
North America	9	US, United States of America (9)
Central America	26	CU, Cuba (25); HN, Honduras (1)
South America	245	AR, Argentine (8); BR, Brazil (234); UY, Uruguay (2); VE, Venezuela (1)
Asia	366	CN, China (7); IN, India (355); KR, South Korea (2); MM, Myanmar (1); PH, Philippines (1)
Northern Europe	106	DK, Denmark (21); FI, Finland (6); NO, Norway (15); SE, Sweden (64)
Western Europe	67	BE, Belgium (34); FR, France (7); GB, Great Britain (3); LU, Luxembourg (3); NL, Netherlands (8); DE, Germany (7); AT, Austria (3); CH, Switzerland (2)
Southern Europe	92	IT, Italy (22); PT, Portugal (28); ES, Spain (26); GR, Greece (3); CY, Cyprus (8); IL, Israel (5)
Eastern Europe	53	PL, Poland (2); CZ, Czech Republic (11); RO, Romania (34); Russia (1); SK, Slovakia (1); UA, Ukraine (3); GE, Georgia (1)
West Africa	63	GA, Gabon (1); GQ, Equatorial Guinea (1); ML, Mali (1); NG, Niger (4); SN, Senegal (56)
East Africa	188	BI, Burundi (91); UG, Uganda (15); TZ, Tanzania (78); KE, Kenya (4)
Horn of Africa	118	ET, Ethiopia (98); ER, Eritrea (2); SD, Sudan (9); DJ, Djibouti (1); SO, Somalia (1); YE, Yemen (7)
Central Africa	644	ZM, Zambia (626); CD, Democratic Republic of Congo (18)
Southern Africa	328	BW, Botswana (133); ZW, Zimbabwe (27); MZ, Mozambique (97); MW, Malawi (71)
South Africa	731	ZA, South Africa (684); SZ, Swaziland (47)
Outgroup	35	Subtype A1 (3); A2 (3); B (4); D (4); F1 (4); F2 (4); G (4); H (4); J (3); K (2)

Supplementary Table S2. Grouping of countries into regions and subcontinents, for the 3,036 (+ 35 outgroup) strains of the worldwide analysis of HIV-1C.

A	P	Cov (%)	Sz	Ps	Sz/Df	Tt	Df	Sl	Sg	Dv	Sl/Dv	Sg/Dv	Sp	Spg
Asia	2	96	**175.500**	**1.5**	**17.643**	184.500	7.500	0.004	0.020	0.069	0.061	0.296	**0.773**	0.829
CentralAfrica	2	78	**250.000**	**2.0**	**2.327**	1575.000	120.500	0.008	0.011	0.104	0.077	0.110	**0.879**	0.879
CentralAmerica	1	65	**17.000**	**2.0**	**17.000**	18.000	1.000	0.010	0.020	0.089	0.109	0.220	**0.838**	0.838
EastAfrica	1	73	**137.000**	**2.0**	**3.806**	574.000	36.000	0.009	0.026	0.093	0.092	0.281	**0.897**	0.897
EasternEurope	1	23	**12.000**	**2.0**	**-**	12.000	0.000	0.015	0.021	0.067	0.222	0.314	**0.971**	0.971
HornOfAfrica	4	74	**21.750**	**1.8**	**2.748**	44.750	11.500	0.005	0.017	0.061	0.079	0.278	**0.841**	0.862
SouthAfrica	2	46	**169.000**	**2.5**	**14.433**	259.000	15.000	0.005	0.016	0.079	0.059	0.205	**0.886**	0.886
SouthAmerica	1	99	**243.000**	**1.0**	**48.600**	251.000	5.000	0.014	0.035	0.091	0.156	0.386	**0.982**	0.982
SouthernAfrica	2	13	**20.500**	**2.0**	**3.200**	51.000	6.500	0.005	0.017	0.058	0.079	0.286	**0.841**	0.911
WestAfrica	1	52	**33.000**	**1.0**	**-**	33.000	0.000	0.022	0.040	0.070	0.310	0.567	**0.990**	0.990
WesternEurope	1	16	**11.000**	**1.0**	**-**	11.000	0.000	0.017	0.024	0.044	0.395	0.559	**0.971**	0.971

Supplementary Table S3. Summary table for the worldwide analysis of HIV-1C, performed with ACCTRAN. This table provides global, average results for the phylotypes selected during analysis. P is the number of significant phylotypes being selected for each annotation (*e.g.* 2 with Asia). Cov (%) is the coverage of the annotation (*e.g.* 96% with Asia), that is, the percentage of strains with the annotation which belong to one phylotype. All other results are averages; for example, *Sz* = 175.500 for Asia is the average *Size* of both Asia phylotypes (*i.e.* 340 and 11, see Tab. S4). See Table 1 and main text for further details and abbreviations.

Pi	Anc	A	Cov (%)	Sz	Ps	Sz/Df	Tt	Df	Sl	Sg	Dv	Sl/Dv	Sg/Dv	Sp	Spg	AnB
2866	1	Asia	3	**11**<br>10/1000	**1**<br>19/1000	**11.000**<br>0/1000	12	1	0.005	0.017	0.054	0.095	0.322	**0.825**	0.825	-
3321	1	Asia	93	**340**<br>0/1000	**2**<br>18/1000	**24.286**<br>0/1000	357	14	0.002	0.023	0.083	0.027	0.271	**0.720**	0.833	-
1	root	CentralAfrica	69	**442**<br>0/1000	**2**<br>686/1000	**2.018**<br>16/1000	3032	219	0.012	0.012	0.131	0.095	0.095	**0.871**	0.871	-
4179	1	CentralAfrica	9	**58**<br>0/1000	**2**<br>686/1000	**2.636**<br>3/1000	118	22	0.005	0.010	0.078	0.058	0.125	**0.887**	0.887	SouthAfrica<br>SouthernAfrica
5077	5063	CentralAmerica	65	**17**<br>0/1000	**2**<br>0/1000	**17.000**<br>0/1000	18	1	0.010	0.020	0.089	0.109	0.220	**0.838**	0.838	-
4844	1	EastAfrica	73	**137**<br>0/1000	**2**<br>0/1000	**3.806**<br>0/1000	574	36	0.009	0.026	0.093	0.092	0.281	**0.897**	0.897	-
789	1	EasternEurope	23	**12**<br>0/1000	**2**<br>0/1000	**∞**<br>0/1000	12	0	0.015	0.021	0.067	0.222	0.314	**0.971**	0.971	SouthernAfrica
183	1	HornOfAfrica	30	**35**<br>0/1000	**2**<br>0/1000	**1.296**<br>0/1000	84	27	0.003	0.007	0.062	0.052	0.112	**0.783**	0.783	-
4913	4844	HornOfAfrica	14	**16**<br>0/1000	**1**<br>0/1000	**5.333**<br>0/1000	19	3	0.005	0.024	0.072	0.068	0.336	**0.851**	0.851	NorthernEurope
5063	4844	HornOfAfrica	8	**10**<br>0/1000	**2**<br>0/1000	**2.000**<br>0/1000	33	5	0.007	0.014	0.058	0.119	0.236	**0.939**	0.939	-
4974	4844	HornOfAfrica	22	**26**<br>0/1000	**2**<br>0/1000	**2.364**<br>0/1000	43	11	0.004	0.022	0.051	0.077	0.429	**0.791**	0.873	NorthernEurope
964	1	SouthAfrica	10	**75**<br>0/1000	**2**<br>951/1000	**18.750**<br>0/1000	80	4	0.005	0.012	0.077	0.062	0.153	**0.886**	0.886	-
1455	1	SouthAfrica	36	**263**<br>0/1000	**3**<br>566/1000	**10.115**<br>0/1000	438	26	0.005	0.021	0.082	0.056	0.257	**0.886**	0.886	-
5204	4844	SouthAmerica	99	**243**<br>0/1000	**1**<br>0/1000	**48.600**<br>0/1000	251	5	0.014	0.035	0.091	0.156	0.386	**0.982**	0.982	-
4578	1	SouthernAfrica	5	**17**<br>0/1000	**2**<br>7/1000	**3.400**<br>0/1000	31	5	0.007	0.024	0.060	0.116	0.395	**0.944**	0.944	SouthAfrica
1488	1455	SouthernAfrica	7	**24**<br>0/1000	**2**<br>7/1000	**3.000**<br>0/1000	71	8	0.002	0.010	0.057	0.042	0.177	**0.738**	0.878	-
398	1	WestAfrica	52	**33**<br>0/1000	**1**<br>0/1000	**∞**<br>0/1000	33	0	0.022	0.040	0.070	0.310	0.567	**0.990**	0.990	-
4310	4179	WesternEurope	16	**11**<br>0/1000	**1**<br>0/1000	**∞**<br>0/1000	11	0	0.017	0.024	0.044	0.395	0.559	**0.971**	0.971	-

Supplementary Table S4. Detailed table for the worldwide analysis of HIV-1C, performed with ACCTRAN. See Table 1 and main text for explanations and abbreviations. In this analysis, some of the links between a phylotype and its ancestor phylotype are indirect, meaning that along the path from the ancestor root (annotated with *A*) to the root of the phylotype (annotated with *B*, possibly equal to *A*) some nodes have incompatible parsimony-based annotation sets (*i.e.* $\{A, B\} \cap S = \emptyset$, where *S* is the annotation set). For example, the path between phylotypes 4179 and 1 (both annotated with CentralAfrica) contains nodes annotated with SouthernAfrica and SouthAfrica, possibly indicating a symmetrical flow and a return of the epidemic to central Africa from southern regions (see main text for discussion).

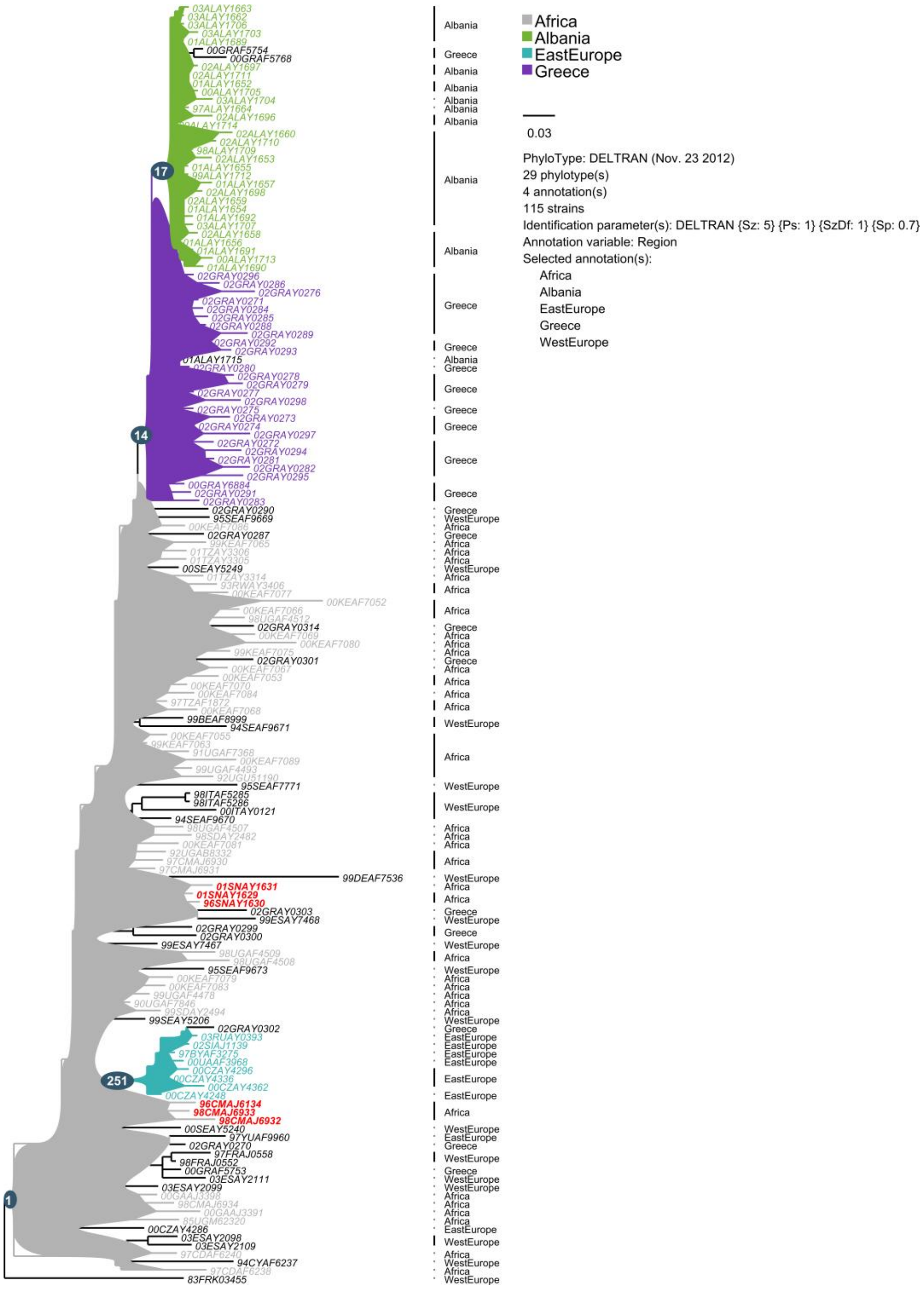

Supplementary Figure S1. Background phylogeny with DELTRAN for the HIV-1A, Albania study. The only difference with ACCTRAN is the presence of the two 3-taxon groups colored in red, which are here inside the large ancestral Africa phylotype. This difference comes from the fact that DELTRAN has less ancestral annotation ambiguities, a general property of this option, compared to ACCTRAN. See note to Figure 3 and main text for further details and explanations.

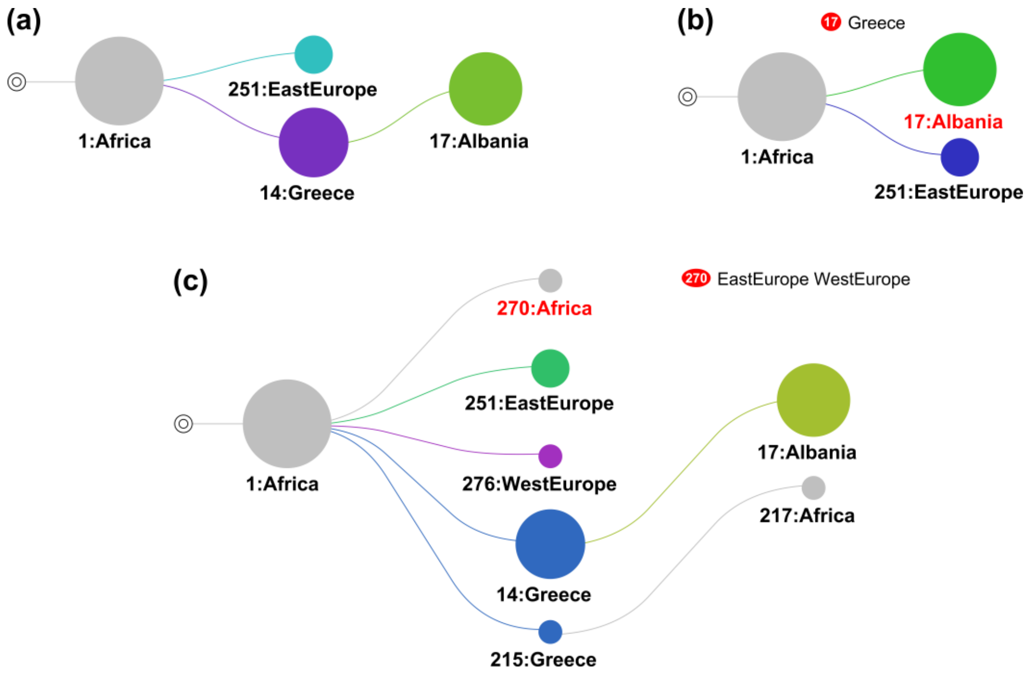

Supplementary Figure S2. Stability of the results regarding parameter values for the HIV-1A, Albania study. The results shown in Figure 3 were obtained with ACCTRAN, *Size* ≥5, *Persistence* ≥1, *Size*/*Different* ≥1 and *Support* ≥0.70; 1,000 shuffles were performed and phylotypes with p-values ≤1% were selected. These selection criteria and thresholds correspond to the default parameter setting of PhyloType. These (minimal) constraints should be relaxed with care, otherwise artifactual results may occur. However, in this study the results appeared to be very stable regarding parameter values. We varied each of the selection parameters independently and checked the phylotype map. The phylotype map (a) is the same as that of Figure 3; it is obtained with 2≤ *Size* ≤8, 1≤ *Persistence* ≤2, 0.5≤ *Size*/*Different* ≤2, and 0.5≤ *Support* ≤0.85. As expected, with *Size* >8 the East Europe phylotype is no longer found, as it contains only 8 members. The same holds true with *Persistence* =3, which imposes strong tree balance constraints. More surprising is the stability of the results regarding aLRT SH-like support values, as the same phylotypes are found, even with *Support* =0. However, we strongly recommend requiring that phylotypes correspond to well-supported clusters. With very high support, some phylotypes are inevitably discarded; here, the Greece phylotype (with support of 0.88, Tab. 1) disappears first, as shown in map (b). Meaningless results were obtained (as expected) when combining low *Size* thresholds with loose (high) p-values. For example, map (c) was obtained with *Size* ≥3, p-value ≤100% (*i.e.* no shuffling-based selection), and all other parameters as in (a); it contains dubious phylotypes, as is Africa (no. 217), descending from Greece with only 3 members and p-value ≈100%. This illustrates (if needed) the importance of using the shuffling procedure and stringent p-values.

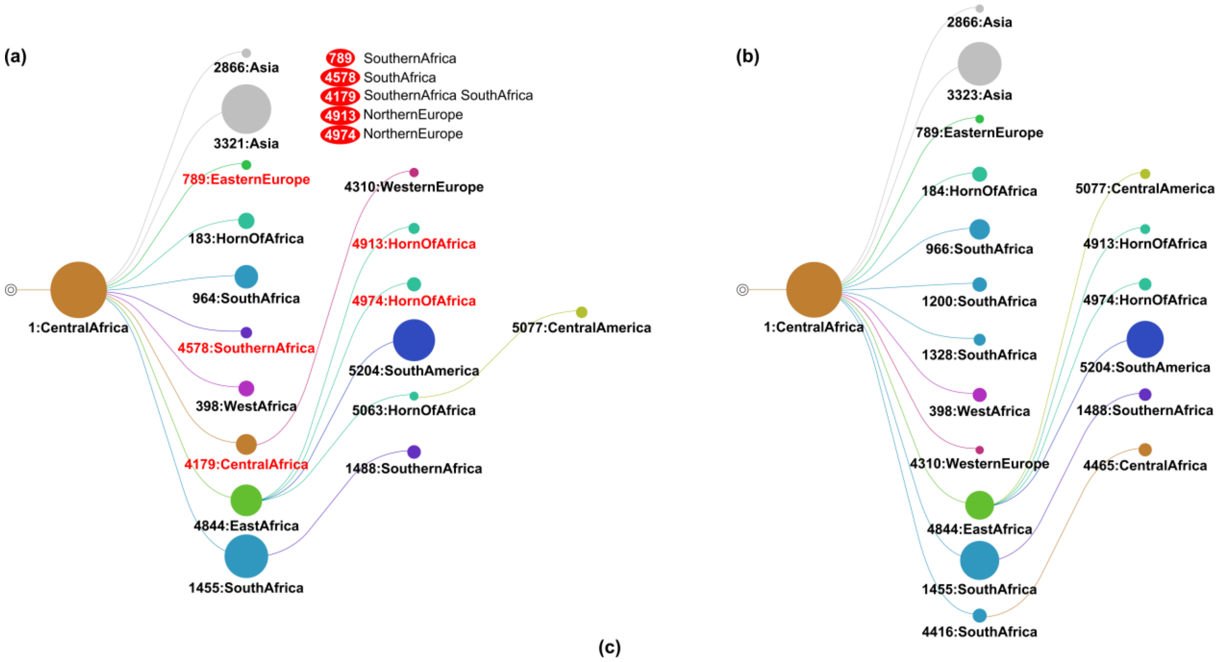

(c)

X = (a)		Y = (b)		#Xi	#Yi	#Xi∩Yi	#Xi∪Yi	∩/∪	Same
Pi	A	Pi	A						origin
5204	SouthAmerica	5204	SouthAmerica	243	243	243	243	1.00	yes
398	WestAfrica	398	WestAfrica	33	33	33	33	1.00	yes
4974	HornOfAfrica	4974	HornOfAfrica	26	26	26	26	1.00	-
5077	CentralAmerica	5077	CentralAmerica	17	17	17	17	1.00	no
4913	HornOfAfrica	4913	HornOfAfrica	16	16	16	16	1.00	-
789	EasternEurope	789	EasternEurope	12	12	12	12	1.00	-
2866	Asia	2866	Asia	11	11	11	11	1.00	yes
4310	WesternEurope	4310	WesternEurope	11	11	11	11	1.00	yes
3321	Asia	3323	Asia	340	337	337	340	0.99	yes
1455	SouthAfrica	1455	SouthAfrica	263	269	263	269	0.98	yes
964	SouthAfrica	966	SouthAfrica	75	72	72	75	0.96	yes
4844	EastAfrica	4844	EastAfrica	137	145	137	145	0.94	yes
1488	SouthernAfrica	1488	SouthernAfrica	24	26	24	26	0.92	yes
183	HornOfAfrica	184	HornOfAfrica	35	38	33	40	0.83	yes
1	CentralAfrica	1	CentralAfrica	442	551	442	551	0.80	yes
4179	CentralAfrica	1	CentralAfrica	58	551	58	551	0.11	-
4578	SouthernAfrica			17	0	0	17	0.00	-
5063	HornOfAfrica			10	0	0	10	0.00	-
		1200	SouthAfrica	0	35	0	35	0.00	-
		4416	SouthAfrica	0	33	0	33	0.00	-
		4465	CentralAfrica	0	29	0	29	0.00	-
		1328	SouthAfrica	0	24	0	24	0.00	-
**All X versus all Y**				**1770 (58.3%)**	**1928 (63.5%)**	**1735**	**1963**	**0.88**	

Supplementary Figure S3. Comparison of ACCTRAN (a) and DELTRAN (b) analyses. The phylotype map (a) is the same as Figure 4; (b) was obtained with the same parameter values but using DELTRAN instead of ACCTRAN. Table (c) reports the similarities and dissimilarities between both analyses. 1,770 and 1,928 sequences are covered by (a) and (b) respectively, corresponding to 18 vs. 19 phylotypes. All phylotypes from (a), denoted as Xi, and (b), denoted as Yi, are displayed with their respective sizes (#Xi and #Yi). Two X and Y phylotypes are "related" (on the same line) when they share common sequences. As we use the same annotation set in (a) and (b), related phylotypes have the same annotation (not true in Supp. Fig. S4 and S5 where the annotation sets are different). We report the size of the

intersection (#Xi∩Yi) and union (#Xi∪Yi) of related phylotypes, and measure their similarity using the intersection/union ratio (∩/∪). A value of 1.0 means that both phylotypes cover the same set of sequences and are identical, while a value close to 0.0 means that their sequence sets are almost disjoined. We see that: 8 (among 18-19) phylotypes are identical; 5 are closely related (∩/∪ > 0.9, with one phylotype included in the other); phylotypes 183(a) and 184(b) are also very similar (0.83, but without inclusion); the large phylotype 1(b) is split into two phylotypes 1(a) and 4179(a); 6 small phylotypes are found by one analysis but not the other, corresponding to a total of 148 sequences. The same measure (∩/∪) is used to assess the global similarity of both analyses, that is, 0.88. It must be noted that sequences covered by ACCTRAN are nearly all (with 35 exceptions) covered by DELTRAN, meaning that both analyses agree fully, but that DELTRAN is more resolved than ACCTRAN. This finding is explained by the fact that DELTRAN resolves more ancestral ambiguities than ACCTRAN. In the last column we compare the origins of phylotypes: "yes" means that the origins of Xi and Yi are related, or are both equal to the map root; "no" indicates that their origins are disjoined; and " - " is used to express that we cannot answer the question (*i.e.* when a phylotype in an analysis is not recovered in the other, or when an origin is indirect and uncertain, *e.g.* (a) no. 4974). We see that 11 (among 18-19) origins are the same for ACCTRAN and DELTRAN, while only 1 differs (corresponding to sequences from Cuba, see text). Both analyses are thus very similar overall, but disagree on some specific points requiring further examination and studies. The PhyloType web interface includes tools for performing comparative analyses such as those described above.

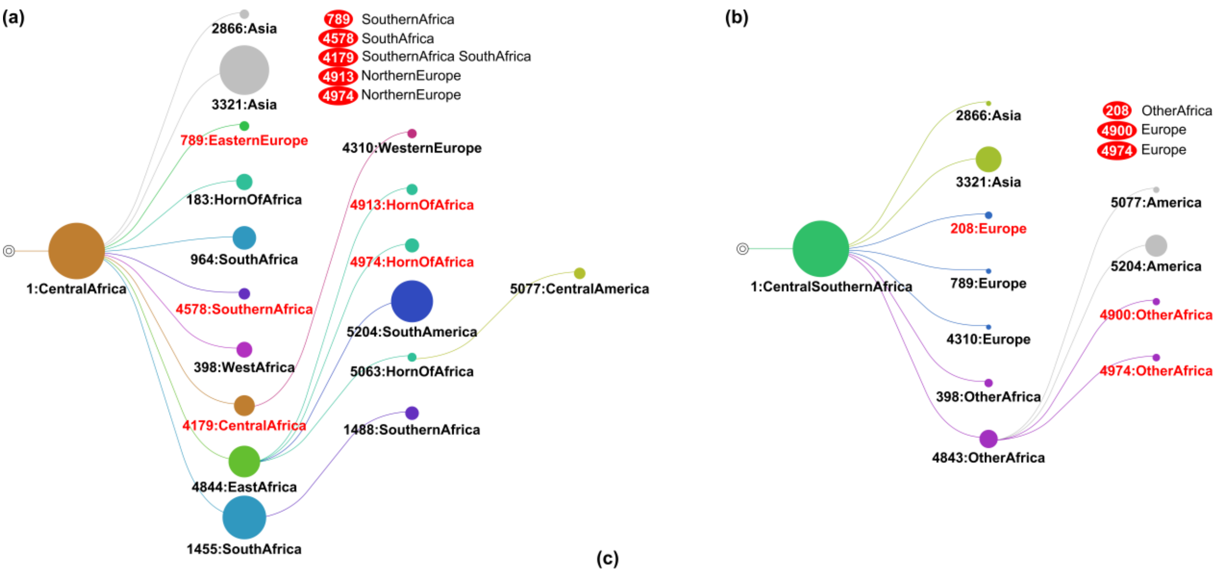

(c)

X = (a)		Y = (b)		#Xi	#Yi	#Xi∩Yi	#Xi∪Yi	∩/∪	Same
Pi	A	Pi	A						origin
3321	Asia	3321	Asia	340	340	340	340	1.00	yes
5204	SouthAmerica	5204	America	243	243	243	243	1.00	yes
398	WestAfrica	398	OtherAfrica	33	33	33	33	1.00	yes
4974	HornOfAfrica	4974	OtherAfrica	26	26	26	26	1.00	-
5077	CentralAmerica	5077	America	17	17	17	17	1.00	yes
789	EasternEurope	789	Europe	12	12	12	12	1.00	-
2866	Asia	2866	Asia	11	11	11	11	1.00	yes
4310	WesternEurope	4310	Europe	11	11	11	11	1.00	yes
4844	EastAfrica	4843	OtherAfrica	137	167	137	167	0.82	yes
5063	HornOfAfrica	4843	OtherAfrica	10	167	10	167	0.06	-
4913	HornOfAfrica	4900	OtherAfrica	16	24	16	24	0.67	-
1	CentralAfrica	1	CentralSouthernAfrica	442	1659	442	1659	0.27	yes
1455	SouthAfrica	1	CentralSouthernAfrica	263	1659	260	1662	0.16	-
964	SouthAfrica	1	CentralSouthernAfrica	75	1659	75	1659	0.05	-
4179	CentralAfrica	1	CentralSouthernAfrica	58	1659	58	1659	0.03	-
1488	SouthernAfrica	1	CentralSouthernAfrica	24	1659	24	1659	0.01	-
4578	SouthernAfrica	1	CentralSouthernAfrica	17	1659	17	1659	0.01	-
183	HornOfAfrica			35	0	0	35	0.00	-
		208	Europe	0	25	0	25	0.00	-
All X versus all Y				1770 (58.3%)	2568 (84.6%)	1732	2606	0.66	

Supplementary Figure S4. Results using (sub)continent annotations for the worldwide HIV-1C study. The phylotype map (a) is the same as Figure 4; map (b) was obtained with the same parameter values and ACCTRAN options as (a), but using broad annotations grouping countries into continents (America, Asia, Europe) and African subcontinents (CentralSouthernAfrica, corresponding to Central, South and Southern Africa in the original (a) analysis, and OtherAfrica for the rest); table (c) compares (a) and (b) analyses and was obtained as explained in the note to Fig. S3. The only difference with Fig. S3 involves the comparison of origins, as different annotation sets are used in (a) and (b): when Xi and Yi are related but the origin of Xi is also related to Yi, then it is not possible to compare the origins of Xi and Yi. For example, phylotype 4844(a) (EastAfrica) is the origin of phylotype 5063(a) (HornOfAfrica), but both are related to phylotype 4843(b) (OtherAfrica) and, hence, the origin of 5063(a) (HornOfAfrica) cannot be compared to the origin of 4834(b) (OtherAfrica);

however, the origins of 4844(a) (EastAfrica) and 4843(b) (OtherAfrica) are comparable (and identical). To summarize the results in table (c): all origins in (a) and (b) are the same or not comparable; 2 small phylotypes are found in one analysis but not the other (60 sequences in total); apart from these 2 phylotypes, all phylotypes of (a) are included (1455(a) is nearly included) in a phylotype of (b), meaning that both analyses are fully compatible; (sub)continent analysis (b) covers many more sequences than (a) (85% vs. 58%), but does not provide much information. Basically, all these results are expected since annotations in (b) are obtained by grouping the annotations in (a).

(a)

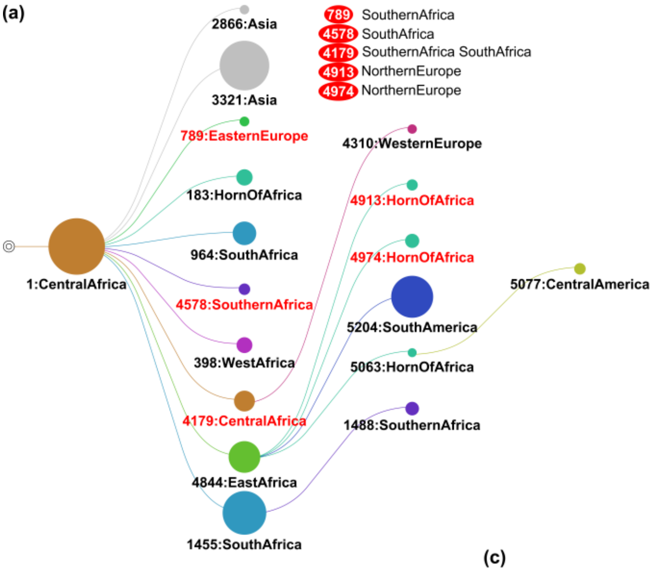

(b)

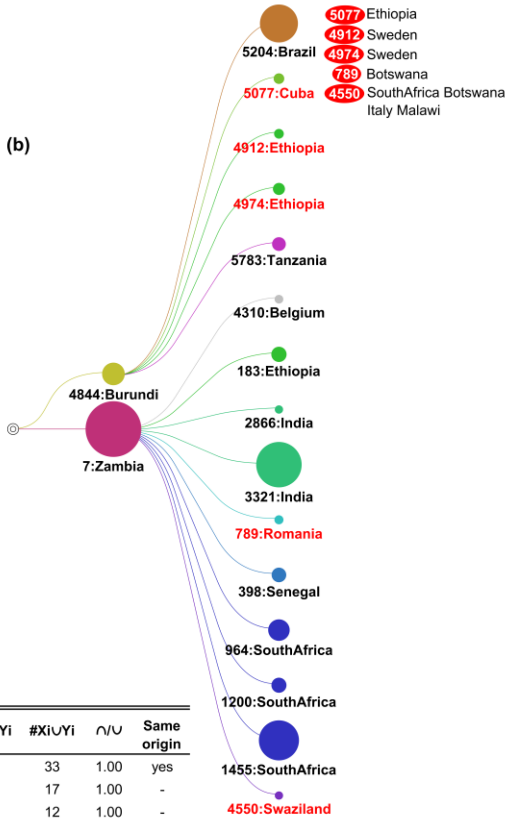

(c)

X = (a) Pi	A	Y = (b) Pi	A	#Xi	#Yi	#Xi∩Yi	#Xi∪Yi	∩/∪	Same origin
398	WestAfrica	398	Senegal	33	33	33	33	1.00	yes
5077	CentralAmerica	5077	Cuba	17	17	17	17	1.00	-
789	EasternEurope	789	Romania	12	12	12	12	1.00	-
4310	WesternEurope	4310	Belgium	11	11	11	11	1.00	yes
1455	SouthAfrica	1455	SouthAfrica	263	260	260	263	0.99	yes
3321	Asia	3321	India	340	335	335	340	0.99	yes
964	SouthAfrica	964	SouthAfrica	75	73	73	75	0.97	yes
5204	SouthAmerica	5204	Brazil	243	233	233	243	0.96	yes
2866	Asia	2866	India	11	10	10	11	0.91	yes
4974	HornOfAfrica	4974	Ethiopia	26	22	22	26	0.85	-
183	HornOfAfrica	183	Ethiopia	35	36	32	39	0.82	yes
1	CentralAfrica	7	Zambia	442	506	426	522	0.82	yes
4179	CentralAfrica	7	Zambia	58	506	58	506	0.11	-
4913	HornOfAfrica	4912	Ethiopia	16	14	13	17	0.76	-
4844	EastAfrica	4844	Burundi	137	81	81	137	0.59	no
4844	EastAfrica	5783	Tanzania	137	29	29	137	0.21	-
1488	SouthernAfrica			24	0	0	24	0.00	-
4578	SouthernAfrica			17	0	0	17	0.00	-
5063	HornOfAfrica			10	0	0	10	0.00	-
		1200	SouthAfrica	0	35	0	35	0.00	-
		4550	Swaziland	0	10	0	10	0.00	-
	All X versus all Y			1770 (58.3%)	1717 (56.6%)	1645	1842	0.89	

Supplementary Figure S5. Results using country annotations for the worldwide HIV-1C study. The phylotype map (a) is the same as that in Figure 4; map (b) was obtained with ACCTRAN and the same parameter values as (a), but using the original annotation corresponding to the 60 countries represented in this study; table (c) compares (a) and (b) maps (see notes to Fig. S3 and S4 for explanations). To summarize: both analyses are closely related (0.89), but (as expected) the country analysis (b) covers less sequences than (a) (56.6% vs. 58.3%); 5 small phylotypes are found in one analysis but not the other (96 sequences in total); apart from these 5 phylotypes, most (b) phylotypes are (nearly) included in (a)

phylotypes (the unique exception concerns CentralAfrica phylotypes 1(a) and 4179(a), which are nearly included in the large Zambia phylotype 7(b)); the main difference concerns the origin-epicenter of the epidemic, which analysis (b) is unable to establish because the DRC sequences (all sampled near the Zambian border) are not grouped with the ones from Zambia. Here we see the interest of grouping annotations to avoid losing the information brought by poorly represented annotation values (*e.g.* DRC, 18 sequences only, but close to the phylogeny root).

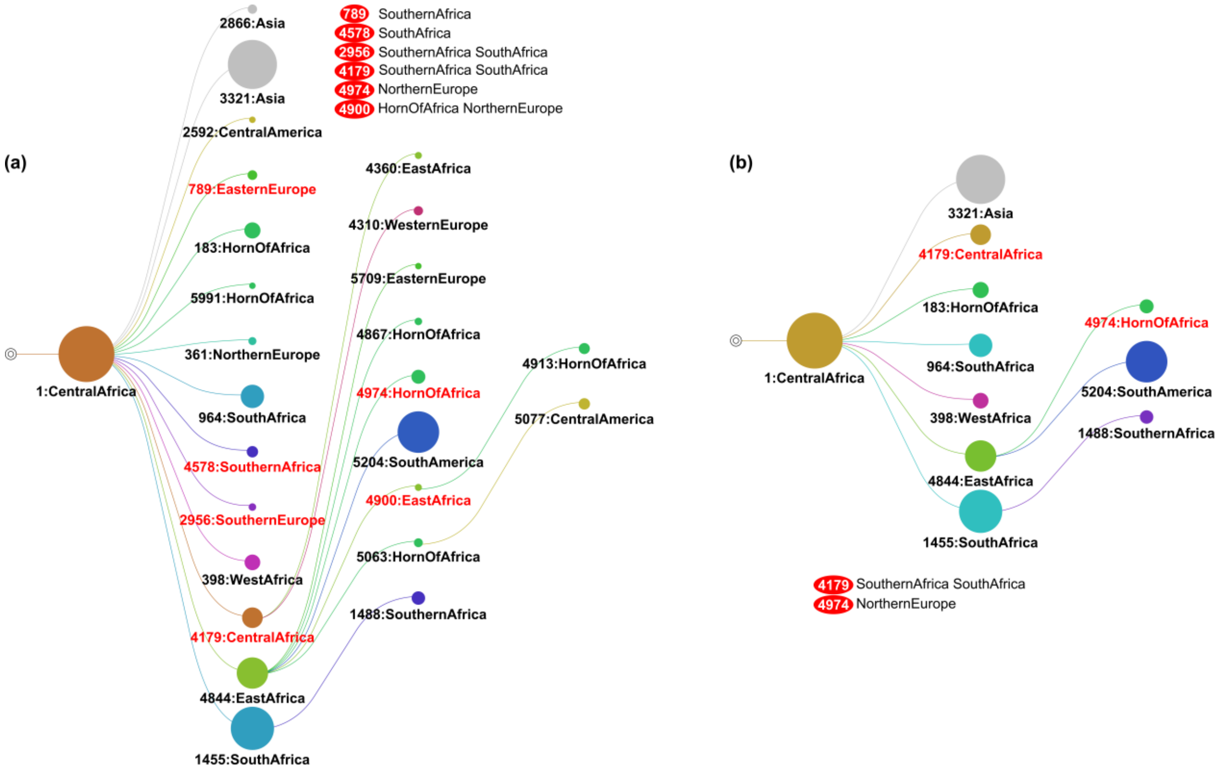

Supplementary Figure S6. Stability of the results re. *Size* value for the worldwide HIV-1C study. Phylotype map (a) was obtained using the same options as that in Figure 4, but *Size* ≥5 (instead of 10). The global similarity with the original map (Fig. 4) is 0.97. As expected, all (18) phylotypes from the original analysis are recovered and identical in both analyses. Moreover, 8 additional, small phylotypes are found (nos. 361, 2956, 4867, 4900, 4360, 5991, 5709, 2592) covering ~1.5% of the sequences. Phylotype map (b) was obtained with *Size* ≥ 20. The global similarity with original analysis is high (0.95): 11 phylotypes remain identical, but 7 phylotypes are lost, corresponding to ~3.5% of the sequences. Overall we see that (as expected) we have more or less phylotypes depending on the *Size* threshold, but the basic results remain identical. The desired level of detail will depend on the user's choices. Here, with more than 3,000 sequences, small phylotypes with ~5 sequences may not be very relevant and a selection threshold of 10 seems appropriate.

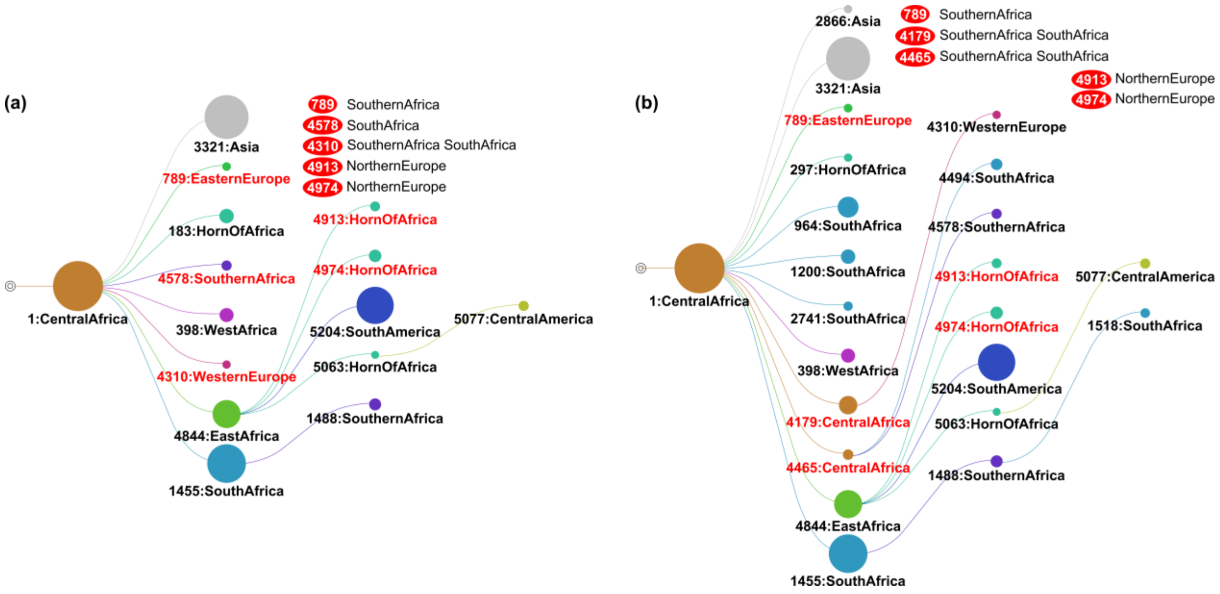

Supplementary Figure S7. Stability of the results re. *Size/Different* value for the worldwide HIV-1C study. Phylotype map (a) was obtained with the same parameter values and options as that in Figure 4, but *Size/Different* ≥0.5 (instead of 1.0). The global similarity with the original map (Fig. 4) is 0.92, and 15 phylotypes (among 18) are recovered and identical in both analyses. However, 3 large phylotypes are lost (nos. 964, 4179, 2866), covering ~5% of the sequences. This unexpected finding is explained by the fact that with a low *Size/Different* threshold, the probability is non-zero of finding large phylotypes by chance (high *Size* value) containing a relatively high number of exceptions (*Different* criterion). This impacts the *Size*-based, shuffling p-values, and some of the original phylotypes in Fig. 4 are discarded because they appear to be non-significant. Phylotype map (b) was obtained with *Size/Different* ≥2.0. The global similarity with the original map (Fig. 4) is 0.93; 17 phylotypes (among 18) are recovered and identical in both analyses; 1 phylotype (no. 183) in Fig. 4 is recovered again (no. 297) in (b), but with low similarity. Moreover, 5 additional phylotypes are found (nos. 1200, 4494, 4465, 1518, 2741), covering ~2% of the sequences. Here we see the opposite effect of that which we observed with a threshold of 0.5: raising the *Size/Different* value renders significant some of the phylotypes that do not appear to be so with lower values. However, when using values that are too large, some phylotypes are lost and split into a number of small phylotypes (not shown). Globally, the results are relatively stable between 0.5 and 2.0, meaning that the *Size/Different* threshold is relatively easy to tune, keeping in mind that it represents the fraction of exceptions that we accept in phylotypes. We cannot admit phylotypes with too many exceptions (low threshold), but being too stringent (high threshold) will result in analyses lacking information with no, or just a few, phylotypes covering a small fraction of sequences.
